# Late Feeding Tube Dependency in Head and Neck Cancer Patients Treated with Definitive Radiation Therapy and Concurrent Systemic Therapy

**DOI:** 10.7759/cureus.7683

**Published:** 2020-04-15

**Authors:** Cole Friedes, Jessica Klingensmith, Nana Nimo, Jessica Gregor, Ryan Burri

**Affiliations:** 1 Medicine, University of Central Florida College of Medicine, Orlando, USA; 2 Family Medicine, St. Petersburg General Hospital, St. Petersburg, USA; 3 Medicine, University of South Florida Morsani College of Medicine, Tampa, USA; 4 Speech Pathology, Cleveland Clinic Florida, Weston, USA; 5 Radiation Oncology, C.W. Bill Young Veterans Affairs Medical Center, Bay Pines, USA

**Keywords:** peg tube, swallowing, dysphagia, chemoradiation, peg dependency, veterans affairs, head and neck cancer

## Abstract

Objective

The study aimed to evaluate the impact of late swallowing dysfunction leading to percutaneous endoscopic gastrostomy (PEG) tube dependence on the overall survival (OS) in a cohort of locally advanced head and neck cancer patients treated and cured with definitive radiotherapy (RT) and concurrent systemic therapy (CST).

Materials and methods

A total of 62 patients with locally advanced head and neck cancer were included in the analysis based on the following selection criteria: stage III, IVA, or IVB disease, treated with definitive RT and CST, no major head and neck surgery, no evidence of local or distant recurrent disease, and at least one post-RT modified barium swallow study. Patients were classified as PEG dependent or PEG independent at the time of the last follow-up. Estimates of OS were calculated using the Kaplan-Meier method. Univariate and multivariate analyses were performed to evaluate the impact of various clinical factors on OS.

Results

The median follow-up was 48 months (range: 7.6-235 months). The five-year OS was 64.3% in the PEG-dependent group and 86.1% in the PEG-independent group (p=0.022). Age over 70 at diagnosis was also associated with poorer OS (p=0.044). On univariate analysis, PEG dependency maintained a significantly worse OS (hazard ratio [HR]: 2.59; 95% confidence interval [CI]: 1.11-5.99, p=0.028). On multivariate analysis, PEG dependency (HR: 4.25; 95% CI: 1.33-13.62; p=0.015), advanced N stage (HR: 4.74; 95% CI: 1.17-19.26, p=0.035), and older age at diagnosis (HR: 4.37; 95% CI: 1.21-15.84; p=0.025) were significantly associated with worse OS.

Conclusions

Late PEG dependency is associated with poor OS in head and neck cancer patients cured with definitive RT and CST. Interventions designed to help head and neck cancer patients maintain swallowing function may result in improved outcomes.

## Introduction

Head and neck cancer is common among the United States Veteran population and is increasingly being managed with organ preservation strategies [1]. Radiotherapy (RT) with or without concurrent systemic therapy (CST) can result in a long-term cure in patients with a variety of primary sites and stages, but late toxicity such as dysphagia and swallowing dysfunction can be debilitating and permanent and lead to dependence on enteral feeding [2-6]. Late percutaneous endoscopic gastrostomy (PEG) tube dependence has been associated with a variety of clinical and tumor factors, but the association between late swallowing dysfunction and overall survival (OS) has not been explored [7-9]. The hypothesis of this retrospective study is that late swallowing dysfunction necessitating PEG support is associated with worse OS in patients with locally advanced head and neck cancer patients cured with definitive RT and CST.

## Materials and methods

Patient and treatment characteristics

The study population included veteran patients referred for a formal swallowing evaluation between 2008 and 2017 to the Speech Pathology Clinic at the Bay Pines VA Healthcare System, C.W. Bill Young Veterans Affairs (VA) Medical Center, after completion of definitive RT and CST for locally advanced head and neck cancer. Informed consent was waived for this Institutional Review Board approved retrospective review of patient medical records.

Patients were included for analysis if they met the following criteria: biopsy-proven squamous cell carcinoma of the head and neck; American Joint Committee on Cancer (AJCC) 7th Edition stage III, IVA, or IVB disease; minimum follow-up of 90 days after completing RT; at least one post-RT modified barium swallow study; no evidence of disease at the time of the last follow-up; and treated with definitive RT and CST. Patients were excluded from analysis if they had undergone major head and neck surgery including definitive surgical resection or neck dissection, had evidence of local, regional, or distant recurrence of head and neck cancer, or had non-squamous cell carcinoma histology.

CPRS records were reviewed to determine disease status at the last follow-up, PEG dependency, overall length of follow-up, and survival status. Patients were assigned either PEG-dependent or PEG-independent status at the time of the last follow-up.

Statistical analysis

Statistical analyses were performed using Statistical Product and Service Solutions (SPSS) Version 24.0 (IBM Corp., Chicago, Illinois, USA) to assess the relationship between clinical, tumor, and treatment characteristics, and PEG dependence and survival. Estimates of OS were calculated using the Kaplan-Meier method, and differences between groups were estimated with the log-rank test. OS was defined as years from the time of diagnosis to death or the last follow-up. Univariate and multivariate Cox-proportional hazards model was used to evaluate the impact of various clinical factors on OS. The chi-square test was used to assess any differences between PEG-dependent and PEG-independent groups at baseline. Statistical significance was defined as p<0.05.

## Results

Patient cohort

Overall, 62 patients were included in the final study population. Distribution of patient demographics, tumor primary site, and staging is available in Table 1.

**Table 1:** Patient characteristics, tumor primary site, and staging *Continuous variables are displayed as median (range) RT, radiotherapy; MBS, modified barium swallow; AJCC, American Joint Committee on Cancer; IMRT, intensity-modulated radiotherapy; 3D, three-dimensional; PEG, percutaneous endoscopic gastrostomy

Characteristic (n=62)	Frequency (%)
Population characteristics	
Median age (years)*	64 (50–94)
Gender	
Male	60 (96.8)
Smoking status	
Current/former	56 (90.3)
History of stroke	
No	58 (93.5)
Median time from RT to MBS (months)*	9.9 (0.2–162)
Tumor primary site	
Oropharynx	31 (50.0)
Base of tongue	16 (25.8)
Tonsil	12 (19.4)
Posterior wall	2 (3.2)
Soft palate	1 (1.6)
Larynx	17 (27.4)
Supraglottic	10 (16.1)
Glottic	7 (11.3)
Hypopharynx	7 (11.3)
Unknown primary	6 (9.7)
Nasopharynx	1 (1.6)
Staging	
AJCC 7th edition stage	
III	15 (24.2)
IVA	45 (72.6)
IVB	2 (3.2)
T stage	
Tx	6 (9.7)
T1	7 (11.3)
T2	19 (30.6)
T3	20 (32.3)
T4a	9 (14.5)
T4b	0 (0.0)
Unknown	1 (1.6)
N stage	
N0	8 (12.9)
N1	13 (21.0)
N2a	2 (3.2)
N2b	21 (33.9)
N2c	15 (24.2)
N3	2 (3.2)
Unknown	1 (1.6)
Treatment characteristics	
Radiation era	
2012 or earlier	30 (48.4)
2013 or later	32 (51.6)
Chemotherapy regiment	
Cisplatin	38 (61.3)
Other	10 (16.1)
Cetuximab	7 (11.2)
Carboplatin	4 (6.5)
Unknown	3 (4.8)
Median radiation dose (cGy)*	7000 (6800–7440)
Radiation schedule	
Conventional	41 (66.1)
Accelerated/hyperfractionated	2 (3.2)
Unknown	19 (30.6)
Radiation modality	
IMRT	36 (58.1)
3D conformal	4 (6.5)
Unknown	22 (35.5)
Received hyperbaric oxygen	
Yes	5 (8.1)
Prophylactic PEG	
Yes	51 (82.2)

The median patient age at the time of treatment was 64 years (range: 50-94 years), with 96.8% of the cohort being males. Most patients were stage IVA at diagnosis (72.6%, n=45), had oropharyngeal tumors (50.0%, n=31), and were treated with platinum-based CST (77.4%, n=48). At the time of analysis, 46 (74.2%) patients were considered PEG dependent and 16 (25.8%) were determined to be PEG independent. Patient, tumor, and treatment characteristics were not significantly different between the PEG- dependent and PEG-independent groups (Table 2).

**Table 2:** Distribution of patient, tumor, and treatment characteristics in PEG-dependent and PEG-independent patients PEG, percutaneous endoscopic gastrostomy; HBO, hyperbaric oxygen; 3D, three-dimensional; IMRT, intensity-modulated radiotherapy

Characteristic	PEG dependent (n = 16)	PEG independent (n = 46)	p-Value
Stage			0.555
III	3	12	
IVA or IVB	13	34	
T stage			0.143
T3 or T4	10	19	
T1 or T2	6	27	
N stage			0.367
N2c or N3	3	14	
N2b or lower	13	32	
Radiation era			0.881
2012 or earlier	8	22	
2013 or later	8	24	
Age at treatment			0.101
71 years or older	5	6	
70 years or under	11	40	
Type of systemic therapy			0.722
Cisplatin	10	31	
Non-cisplatin	6	15	
Hyperbaric oxygen			0.068
Received HBO	3	2	
Did not receive HBO	13	44	
Prophylactic PEG			0.617
Yes	13	38	
No	3	6	
Unknown	0	2	
Radiation modality			0.301
3D conformal	2	2	
IMRT	7	29	
Unknown	7	15	
Tumor primary site			0.477
Larynx/hypopharynx	5	19	
Other	11	27	
Tobacco use			0.590
Yes/former	15	41	
No	1	5	

Factors influencing overall survival

The following clinical information was collected and analyzed by univariate analysis for association with OS: PEG dependence, overall stage, T stage, N stage, radiation era, age at treatment, type of CST, use of hyperbaric oxygen, prophylactic PEG use, tumor primary site, and tobacco use (Table 3).

**Table 3:** Univariate analysis of patient and treatment characteristics associated with OS CI, confidence interval; OS, overall survival; PEG, percutaneous endoscopic gastrostomy; HBO, hyperbaric oxygen

Factor	Hazard ratio (95% CI)	Five-year OS (%)	p-Value
PEG dependence			
PEG dependent	2.59 (1.11–5.99)	64.3	0.028
PEG independent (reference)		86.1	
Stage			
III	1.46 (0.25–1.91)	75.8	0.468
IVA or IVB (reference)		83.0	
T stage			
T3 or T4	1.05 (0.40–2.23)	88.1	0.904
T1 or T2 (reference)		74.0	
N stage			
N2c or N3	0.65 (0.62–3.82)	73.7	0.353
N2b or lower (reference)		83.9	
Radiation era			
2012 or earlier	2.70 (0.09–1.53)	90.0 (four-year OS)	0.155
2013 or later (reference)		80.2 (four-year OS)	
Age at treatment			
71 years or older	2.57 (0.99–6.65)	72.7	0.052
70 years or under (reference)		82.9	
Type of systemic therapy			
Cisplatin	0.87 (0.47–2.81)	82.7	0.752
Non-cisplatin (reference)		75.2	
Hyperbaric oxygen			
Received HBO	0.68 (0.49–4.50)	60.0	0.486
Did not receive HBO (reference)		83.4	
Prophylactic PEG			
Yes	0.52 (0.63–5.85)	79.4	0.248
No (reference)		87.5	
Primary site			
Larynx/hypopharynx	1.16 (0.35–2.09)	75.8	0.740
Other (reference)		83.9	
Tobacco use			
Yes/former	0.78 (0.30–5.62)	80.8	0.734
No (reference)		80.0	

Detailed RT dosimetric information was available for only 25 of the 62 patients; therefore, information on radiation modality and dose was not included in the univariate and multivariate analyses.

On univariate analysis, PEG-dependent patients (hazard ratio [HR]: 2.59; 95% confidence interval [CI]: 1.11-5.99; p=0.028) and patients aged 71 years or older at treatment (HR: 2.57; 95% CI: 0.99-6.65; p=0.052) had worse OS outcomes. No other factors were associated with OS on univariate analysis. On multivariate analysis, the following were recognized to have worse OS: PEG dependency (HR: 4.25; 95% CI: 1.33-13.62; p=0.015), advanced N stage (HR: 4.74; 95% CI: 1.17-19.26; p=0.032), and older age at treatment (HR: 4.37; 95% CI: 1.21-15.84; p=0.014) (Table 4).

**Table 4:** Multivariable Analysis of patient and treatment characteristics associated with survival CI, confidence interval; PEG, percutaneous endoscopic gastrostomy; HBO, hyperbaric oxygen

Factor	Hazard ratio (95% CI)	p-Value
PEG dependence	4.25 (1.33–13.62)	0.015
Lower overall stage	1.06 (0.21–5.45)	0.945
Higher T stage	1.16 (0.44–3.04)	0.763
Higher N stage	4.74 (1.17–19.26)	0.030
Earlier radiation era	0.36 (0.07–1.84)	0.220
Older age at treatment	4.37 (1.21–15.84)	0.025
Cisplatin therapy	1.20 (0.44–3.29)	0.719
HBO use	1.18 (0.31–4.48)	0.808
Prophylactic PEG	3.09 (0.75–12.78)	0.119
Primary tumor site	1.10 (0.29–4.16)	0.884
Tobacco use	0.53 (0.07–3.93)	0.530

**Figure 1:**
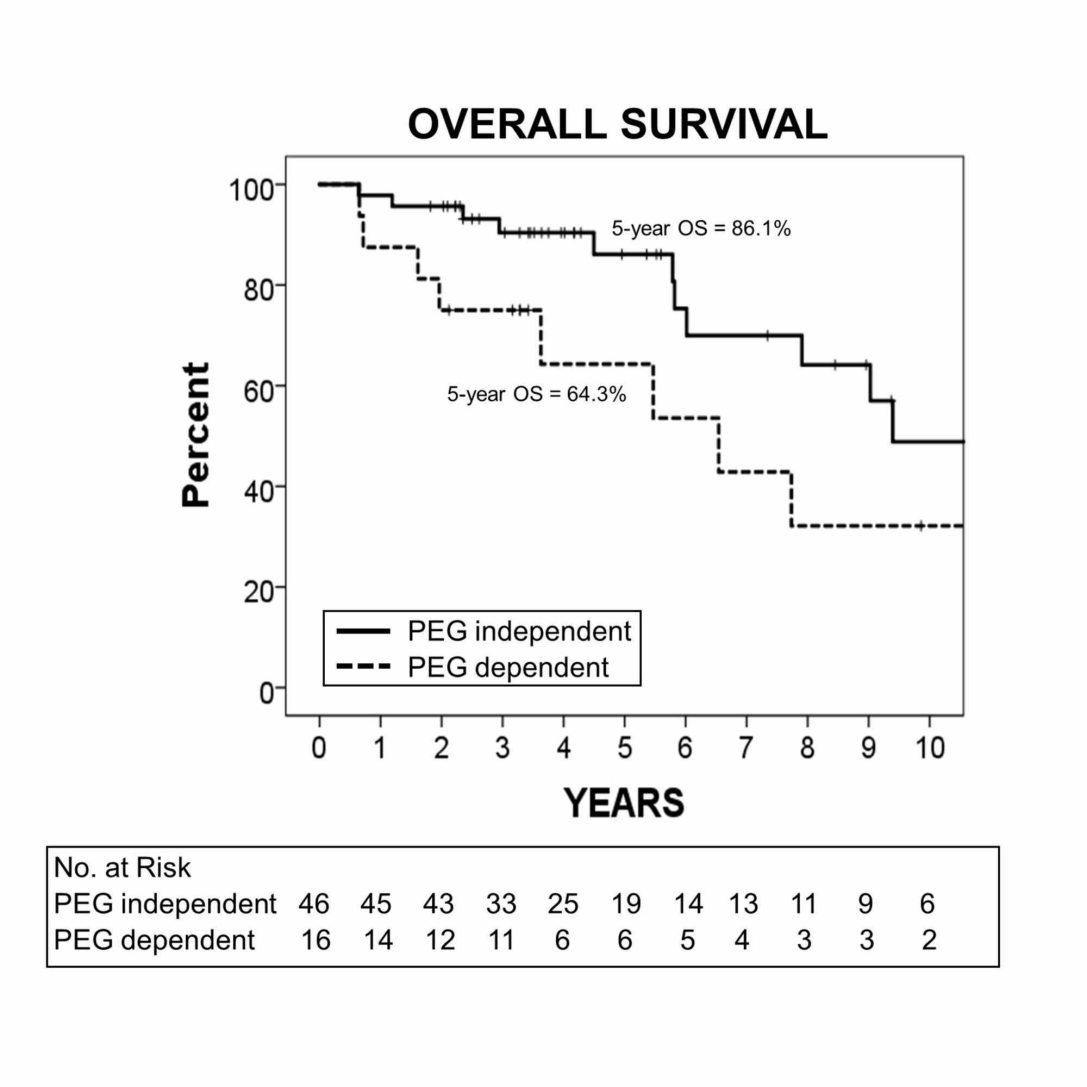
The Kaplan-Meier survival curve showing OS for PEG-dependent (dashed line) and PEG-independent (solid line) patients, p=0.022 (log-rank test) OS, overall survival; PEG, percutaneous endoscopic gastrostomy

The median follow-up after the completion of definitive RT and CST was 48 months (range: 7.6-235 months). There was an observed worsening in OS for PEG-dependent patients (median of 6.54 years [95% CI: 3.62 - NA] with PEG dependence vs. median of 9.39 years [95% CI: 7.90 - NA] with PEG independence). The five-year OS was 64.3% in the PEG-dependent group and 86.1% in the PEG-independent group (p=0.022) (Figure 1). Older age at treatment was also associated with poorer OS (p=0.044, not shown).

## Discussion

In a cohort of patients with non-resected stage III or IVA/B head and neck squamous cell carcinoma definitively cured with RT and CST in the C.W. Bill Young VA Medical Center, we found that PEG dependency was significantly associated with worse OS compared with PEG-independence. Therefore, long-term RT and CST toxicities leading to late feeding tube dependence may have substantial survival implications, and the provider decision for PEG placement should be weighed heavily. Similarly, in accordance with the NCCN (National Comprehensive Cancer Network) guidelines and other literature, prophylactic PEG tubes and enteral feeding should only be employed when unequivocally necessitated [10,11]. The risks of potential PEG tube dependence must be weighed against the nutritional benefits. By the best of our knowledge, this is the first report describing survival outcomes in a PEG-dependent cohort in comparison with their PEG-independent counterparts.

It is well known that organ-preserving strategies used to cure locally advanced head and neck carcinoma may induce significant acute and long-term side effects. Acute toxicities such as skin toxicity or mucositis often temporarily disrupt swallowing function during treatment but are often transient in nature. In contrast, late effects including neuropathy and fibrosis of oropharyngeal musculature may result in permanently disabling dysphagia, necessitating enteral support to reduce the risk of life-threatening pneumonia and support nutritional habits [12-14]. One smaller study found that as a result of late toxicity dysphagia, 66% of the cohort was gastrostomy-dependent [15]. Known predictors of PEG tube placement include patient advanced age, primary tumor site, smoking status, higher T and N stages, body mass index of less than 25, accelerated irradiation fractionation, and use of high-dose chemotherapy [7,9].

While feeding tube dependency is relatively common, few studies have explored the relationship between enteral support and survival [4,5,16,17]. At the time of the last follow-up, 16 (26%) patients of our cohort were found to be feeding tube dependent. In the GORTEC 99-02 trial comparing fractionation schedules for locally advanced head and neck carcinoma, rates of feeding tube placement varied, with 60% in the conventionally fractionated group, 64% in the hyperfractionated, and 70% in the very accelerated RT patients. At the five-year follow-up, 13% of conventionally fractionated patients were feeding tube dependent versus 25% of the very accelerated RT group (p=0.027) [4]. Pooled analyses have also demonstrated a feeding tube dependency rate of 10.3% in a locally advanced cohort [5]. A small retrospective study evaluated the timing of PEG placement and OS and showed that prophylactic PEG use had worse outcomes (HR: 11.62; 95% CI: 1.77-76.47; p=0.011) [17]. However, this study did not evaluate long-term PEG dependency and may thus explain our study results that prophylactic PEG use did not result in worse OS, rather long-term PEG dependency appears to be a key factor in driving survival.

Reducing post-chemoradiation toxicity may improve OS due to decreased PEG tube dependence. The mean dose to swallowing organs significantly predicts long-term dysphagia; ergo, it is possible that intensity-modulated RT (IMRT) may lead to less dysphagia as the technique can spare the pharyngeal constrictor muscles and supraglottic larynx [18,19]. Although not yet clinically validated, the introduction of functional swallowing units (FSUs), defined by hyolaryngeal elevation, tongue base retraction, and tongue motion, is certainly promising. Using these contouring guidelines to mark the delineation of FSUs may reduce toxicity, leading to better outcomes [20,21]. A new randomized control trial is planned to more definitively explore the role of IMRT in reducing dysphagia [22]. Regarding our study, more than half of our cohort received IMRT-based therapy. However, due to unavailable data for 35.5% of patients, radiation modality was not included in our survival analysis. Of the 36 IMRT patients, only 7 (19.4%) were PEG dependent at the last follow-up. Future research may show that patients receiving IMRT have better outcomes due to less PEG dependency.

Similarly, toxicity mitigation through non-treatment altering programs offers promise in the reduction of late dysphagia. Prophylactic swallowing programs have frequently been investigated as an attempt to reduce post-radiation fibrosis and improve dysphagia-related outcomes [23-26]. Carnaby-Mann et al. demonstrated that twice daily swallowing musculature exercises resulted in significantly less dysphagia compared with usual care and sham exercises, whereas van der Molen et al. showed that preventative exercises resulted in less feeding tube dependency compared with other institutional trials [23,25]. Overall, these studies concluded that swallowing programs have a beneficial reduction in acute swallowing issues. However, long-term outcomes and if these programs lead to a reduction in PEG dependence or OS are still unknown. On the other hand, additional retrospective studies have shown that prophylactic PEG tubes often lead to increased esophageal strictures and increased PEG dependence, and do not impact treatment times [10,24,27]. These previous studies suggest that avoidance of prophylactic PEG tubes may improve outcomes, which is reinforced by our findings that PEG dependence is associated with worse OS.

Recently, efforts to de-intensify treatment to reduce significant toxicity while delivering similar efficacy have been explored. A recent analysis of VA patients receiving definitive concurrent chemoradiation for stage III-IVB unresectable head and neck cancer showed that low-dose cisplatin compared with high-dose cisplatin resulted in no change in survival with simultaneous toxicity reduction [28]. Similarly, in an early de-intensification trial, Chera et al. showed that de-intensification of chemoradiotherapy (e.g., with 60 Gy of IMRT and ipsilateral RT for tonsil cancers) resulted in favorable outcomes [6]. In the evolving era of immunotherapy, we anticipate the incorporation of immunotherapy into the standard of care for locally advanced and advanced head and neck carcinomas. We eagerly anticipate the results of two clinical trials evaluating the role of pembrolizumab in locally advanced head and neck malignancies (NCT03040999) and in patients who are cisplatin-ineligible due to significant toxicity (NCT02609503).

We acknowledge that there are limitations of a small cohort-based, retrospective analysis. First, our study did not have the means to assess human papillomavirus (HPV) or p16 status. These factors offer a prognostic benefit to patients with squamous cancers of the head and neck and can significantly alter outcomes [29]. Specific staging for HPV positive oropharyngeal cancer has been adopted in the AJCC 8th edition staging guidelines; therefore, a more careful stratification of non-HPV or HPV-related cancers in patients with PEG dependence must be assessed. Due to the nature of our study, we are unaware of the indications for original PEG tube placement or for the nature of speech pathology referral. Protocols for PEG placement often vary at clinical institutions, and the uncertainty as to why each PEG tube was used offers confounding factors in our study. Also, our study does not include data regarding nasogastric tubes, which are sometimes used as an alternative to PEG tubes. Finally, we acknowledge that our survival outcomes are far superior to landmark and other similar trials. The five-year OS rates reported in these trials range from approximately 20 to 45%, depending on the primary site [28,30]. The actuarial five-year OS for this patient cohort was higher (84.1% for PEG-independent patients and 64.3% for PEG-dependent patients) because only patients who had no evidence of disease at the time of the last follow-up were included in the analysis. The study was designed in this manner to decrease confounding on OS and further strengthen the relationship between late swallowing dysfunction, PEG dependency, and OS.

In the future, we hope to expand our analyses from our single institution. We aim to use the VA Corporate Data Warehouse (CDW) to achieve this goal. The CDW pools medical records from VA centers and other government databases across the nation to afford the opportunity for large-scale data mining and allow access to multicenter informatics. We anticipate that expanding our study cohort may offer more insight into the details that explain our findings.

## Conclusions

In conclusion, our data suggest that PEG dependence is significantly associated with worse OS, further supporting the fact that minimizing RT and CST side effects drastically improves patient care. Providers should carefully consider the use of enteral feeding and recognize the potential risks associated with late feeding tube dependency.
